# Crosstalk Between Autophagy and Hypoxia-Inducible Factor-1α in Antifungal Immunity

**DOI:** 10.3390/cells9102150

**Published:** 2020-09-23

**Authors:** Tim Quäschling, Dirk Friedrich, George S. Deepe, Jan Rupp

**Affiliations:** 1 Department of Infectious Diseases and Microbiology, University of Lübeck, 23562 Lübeck, Germany; tim.quaeschling@uksh.de (T.Q.); dirk.friedrich@uksh.de (D.F.); 2 Division of Infectious Diseases, University of Cincinnati College of Medicine, Cincinnati, OH 45229, USA; Deepegs@ucmail.uc.edu; 3 German Center for Infection Research (DZIF), Partner Site Hamburg-Lübeck-Borstel-Riems, 23562 Lübeck, Germany

**Keywords:** autophagy, LC3-associated phagocytosis (LAP), fungal immunity, hypoxia-inducible factor-1α (HIF-1α), microtubule-associated protein 1A/1B-light chain 3 (LC3), *Histoplasma capsulatum*, *Aspergillus fumigatus*, *Cryptococcus neoformans*, *Candida albicans*, xenophagy

## Abstract

Modern medicine is challenged by several potentially severe fungal pathogens such as *Aspergillus fumigatus*, *Candida albicans*, or *Histoplasma capsulatum*. Though not all fungal pathogens have evolved as primary pathogens, opportunistic pathogens can still cause fatal infections in immuno-compromised patients. After infection with these fungi, the ingestion and clearance by innate immune cells is an important part of the host immune response. Innate immune cells utilize two different autophagic pathways, the canonical pathway and the non-canonical pathway, also called microtubule-associated protein 1A/1B-light chain 3 (LC3) -associated pathway (LAP), to clear fungal pathogens from the intracellular environment. The outcome of autophagy-related host immune responses depends on the pathogen and cell type. Therefore, the understanding of underlying molecular mechanisms of autophagy is crucial for the development and improvement of antifungal therapies. One of those molecular mechanisms is the interaction of the transcription-factor hypoxia-inducible factor 1α (HIF-1α) with the autophagic immune response. During this review, we will focus on a comprehensive overview of the role of autophagy and HIF-1α on the outcome of fungal infections.

## 1. Introduction

During the last years, the significance of autophagy to the innate immune response against fungal infections has become evident. There are 32 identified autophagy-related genes (Atg) conserved across different phylogenies such as plants, mammals, and yeasts, thus, the importance of autophagy for maintaining cell homeostasis is widely accepted [1,2]. Originally recognized as a mechanism to re-introduce cellular components into metabolic pathways, autophagy plays a major role in the innate immune response. There have been two forms of autophagy reported. Both the canonical and the non-canonical autophagy pathways initiate degradation of intracellular components in the lysosome. The canonical pathway is activated through different stimuli including nutritional starvation, low oxygen concentration, infection, or endoplasmic reticulum stress caused by glucose deprivation, as an example [3,4]. Autophagy selectively targets pathogens and is mainly induced when pathogen-associated molecular patterns (PAMPs) and pathogen-induced damage-associated molecular patterns (DAMPs) are recognized by pattern recognition receptors (PPRs) such as Dectin1 or toll-like receptors (TLRs) [5]. Upon pathogen-recognition, the autophagy inhibitor, the mammalian target of rapamycin (mTOR), is blocked and autophagy is induced. This process is followed by the formation of the autophagosome [6].

Concomitant with phagophore nucleation, microtubule-associated protein 1A/1B-light chain 3 (LC3) is recruited to this cellular compartment and, after lipidation of LC3-I to LC3-II, the latter is incorporated into the inner and outer surface of the autophagosome [7]. LC3-II facilitates the fusion of the matured autophagosome with the lysosome to form the autolysosome, and the pathogens are killed through acidification [8]. The impact of autophagy in innate cells extends beyond killing. Regarding dendritic cells (DCs), autophagy is crucial for the presentation of antigens to cluster of differentiation (CD)4^+^T cells via the major histocompatibility complex (MHC) class II molecules, linking the innate to the adaptive immune response [9]. Support for the in vivo importance of autophagy in antigen presentation was revealed in studies in which a deletion of autophagy-related gene (Atg)5 in mouse DCs impaired priming of T cells after infection with herpes simplex virus [10]. Concerning neutrophils, autophagy is vital for the secretion of granules containing antimicrobial and inflammatory proteins [11]. This release is highly interconnected with an event called neutrophil extracellular trap (NET) formation. During this process, neutrophils release intracellular contents such as histones and calprotectin that exert antimicrobial activity [12]. An increasing amount of evidence points to a close interaction between autophagy and NET formation [13]. The latter is highly regulated by production of reactive oxygen species (ROS)which is in turn influenced by autophagy [14]. Neutrophils lacking Atg5 are deficient in NET formation [15]. Conversely, initiation of autophagy leads to an increase in released NETs [16].

Some fungal pathogens, such as *Aspergillus fumigatus*, can be recognized upon first contact with the immune cell and the non-canonical autophagy pathway, also called the LAP-pathway, is triggered. The LAP-pathway initiates the selective ingestion and degradation of fungal cells. To contrast to canonical autophagy, the LAP-pathway does not utilize de-novo formation of the double-membraned autophagosome but, instead, drives the recruitment of LC3-II to the phagosome after phagocytosis [17]. Engaging in cell surface receptor signalling, the LAP-pathway conjugates LC3-II to already existing single-membrane phagosomes [18]. Therefore, the formation of these so-called LAPosomes is independent of the canonical signalling cascade and phagophore-formation inside the immune cell. Interestingly, the initiation of the LAP-pathway inhibits the classical autophagy pathway and vice versa [18].

The LAP pathway starts with the stimulation of the Dectin-1/Spleen tyrosine kinase (Syk) complex [19]. Activation of this complex is followed by the mobilization of the class III phosphatidylinositol 3-kinase (PI3K) complex, formed by Beclin1, vacuolar protein sorting 34(VPS34) kinase, UV radiation resistance-associated gene protein (UVRAG) and Rubicon, to the LAPosome [18]. Localization of the class III PI3K-complex to the LAPosome prompts generation of phosphatidylinositol 3-phosphate (PI3P) and is followed by the stabilization of the nicotinamide adenine dinucleotide phosphate (NADPH) oxidase-2 (NOX2) complex and ROS. The combination of ROS and PI3P leads to the recruitment of the LC3-conjugation machinery. Maturation of the LAPosome is achieved by the binding of LC3 [20]. The LAPosome fuses with the lysosome, forming the phagolysosome and pathogens are killed through acidification. Simultaneous to the initiation of autophagy, recognition of pathogens also leads to the accumulation of transcription factor hypoxia-inducible factor-1α (HIF-1α) via the Syk-pathway and nuclear factor kappa-light-chain-enhancer of activated B cells (NFκB) [21].

HIF-1α is an important mammalian regulator of gene expression during hypoxia, but also plays a key role in the regulation of host immune responses during fungal infections. Several studies show the downstream stimulation of HIF-1α, either by hypoxia or infection [22,23], as well as the exposure to nitric oxide (NO) [24]. Considering non-infected cells, HIF-1α is regulated by the amount of ambient oxygen. Under normoxic conditions (>21% O_2_) HIF-1α is targeted for degradation by the proteasome, while under hypoxic conditions (<6% O_2_) HIF-1α stabilizes and translocates into the nucleus, forming the HIF-1 complex by binding to HIF-1β. To contrast to stabilization of HIF-1α under hypoxic conditions, exposure to NO under normoxic conditions upregulates HIF-1α synthesis without modulating proteasomal degradation [24]. Then, in return, HIF-1α upregulates inducible nitric oxide synthase (iNOS), starting a positive feedback loop [25].

HIF-1 binds to hypoxic response elements (HRE) in the promotor regions of target genes. Liu et al., [26] have stated that induction of the autophagic machinery by infection stabilizes HIF-1α, meaning that even under normoxic conditions a stabilization of HIF-1α can occur. To contrast, several studies have shown the activation of autophagy downstream of HIF-1α [5,27], suggesting a positive feedback loop in which autophagy is needed to generate energy to support the innate immune response while HIF-1α modulates the cell metabolism as part of the immune response. This transcription factor induces a series of alterations in macrophages (MΦ), including increases in inflammatory cytokines, chemokines, and antimicrobial peptides. HIF-1α stabilization is vital for the proper migration of MΦ into inflammatory tissue [28]. Collectively, the data show that HIF-1α regulates a number of key functions of innate cells for them to control infections [29]. During this review, we will focus on role of the LAP-pathway for the innate immune response during fungal infections. We will take a closer look at the impact of HIF-1α regulation on the outcome of antifungal immune responses as part of the LAP-pathway.

## 2. Impact of Autophagic Pathways on Fungal Infections

Known for all microbial infections, the coordination of the first innate immune response and the subsequent adaptive immune response is critical for a successful clearing of infection. Tissue-specific and recruited inflammatory phagocytes, such as macrophages (MΦ) and neutrophils, are the first immune cells to respond to an infection. They are also critical for the killing of fungal pathogens in early stages of the infection. Dendritic cells (DCs) are the link between the innate immune response and the adaptive immune system. They are the professional antigen-presenting cells (APCs) that prime naïve T cells [30] by presenting fungal antigens. Fungi recognized after they enter a cell are targeted and cleared by the canonical pathway. Additionally, fungal pathogens are recognized by cell surface receptors of immune cells and trigger the LC3-associated phagocytosis (LAP)-pathway. Independent of which autophagic pathway is engaged, the process of killing other organisms by autophagy also is known as xenophagy [31].

### LC3-Associated Phagocytosis (LAP)

Receptors such as Dectin-1 or toll-like receptors (TLRs) on the cell surface of innate immune cells often recognize fungal pathogens in the extracellular matrix and initiate LC3-associated phagocytosis (LAP). Unlike the canonical pathways, LAP relies on the Rubicon/Beclin1-complex and nicotinamide adenine dinucleotide phosphate (NADPH) oxidase-2 (NOX2) activation rather than regulation by mechanistic target of rapamycin (mTOR). Upon recognition, fungal pathogens are engulfed by phagocytosis and trigger activation of the Dectin-1/Spleen tyrosine kinase (Syk) complex [32]. Activation of this pathway ultimately results in the generation of reactive oxygen species (ROS) and phosphatidylinositol 3-phosphate (PI3P). The combination of ROS and PI3P lead to the recruitment of the microtubule-associated protein 1A/1B-light chain 3 (LC3)-conjugation machinery consisting of the autophagy-related gene (Atg)5/Atg12/Atg16L1-complex and Atg3, Atg4 and Atg7 [33]. Comparable to the canonical autophagy pathway, LC3-II is incorporated into the outer surface of the LAPosome. Ultimately, LC3-II assists in the fusion of the LAPosome with the lysosome, consequently killing fungi. Studies in dendritic cells (DCs) have shown that the conjugation of LC3-II is crucial for the major histocompatibility complex (MHC) class II presentation of fungal-derived antigens [34]. To contrast to these findings, the presentation of MHC class I molecules is decreased during autophagy [35]. Still, there is evidence that LAP promotes cross-presentation of antigens with MHC class I molecules [36]. Activation of innate immune cells by recognition of fungal pathogens also leads to a change in metabolism. During infection with *Candida albicans* or *Histoplasma capsulatum*, immune cells upregulate anaerobic glycolysis and oxidative phosphorylation [37,38]. To contrast to these findings, immune cells activated by lipopolysaccharide (LPS) only upregulate anaerobic glycolysis while downregulating oxidative phosphorylation. This event also is known to occur under normoxic conditions when initiated by LAP. Accompanying the modulation of the metabolism to increased glycolytic flux is the stabilization of hypoxia-inducible factor-1α (HIF-1α) [23]. This can be seen during activation of innate immune cells by TLR ligands as well as infection by intracellular pathogens [39]. Once HIF-1α stabilizes, a positive feedback loop is activated in which HIF-1α fuels the increase in the glycolytic flux. One reason for the glycolytic switch is the preparation to enter areas of inflammation which are hypoxic. However, the activity HIF-1α is not restricted only to metabolism.

## 3. Autophagy in Antifungal Immunity—Friend or Foe

The widespread and increasing use of biologicals and immunosuppressive therapies facilitate the emergence of potentially severe, fungal infections such as *Aspergillus fumigatus, Candida albicans*, *Cryptococcus neoformans,* and *Histoplasma capsulatum*. Recognition of one of these pathogens mediates the activation of antimicrobial chemokines and cytokines, which then initiate the anti-fungal response. Based on the route of entry, or the way a fungal pathogen is recognized, the immune response can differ. Some fungi, such as *Aspergillus fumigatus,* have developed immune evasion strategies, while others, such as *Histoplasma capsulatum* and *Cryptococcus neoformans,* need the autophagic response for survival and proliferation. Autophagy is an efficient tool for the containment and killing of fungal pathogens. Simultaneously, once a pathogen is phagocytosed, it can no longer be targeted by other immune cells, thus evading the adaptive immune response, for example. Furthermore, they might take advantage of the metabolic process autophagy supplies, utilizing nutrients inside the phagosome that should have been re-introduced into the metabolic cycle. During the following, we will take a look at different fungal pathogens and their interactions with the autophagic machinery.

### 3.1. Aspergillus Fumigatus

*Aspergillus* conidia are engaged by LC3-associated phagocytosis (LAP), but not classical autophagy, putting emphasis on the importance of LAP [18]. *Aspergillus fumigatus* is recognized by Dectin-1 receptors which play a fundamental role in the activation of the LAP pathway. Defective β-glucan recognition, caused by the loss of Dectin-1, produces a higher susceptibility to infection with *A. fumigatus* in mice [40]. Humans with a genetic polymorphism affecting Dectin-1 show a higher susceptibility to aspergillosis [41].

Disrupting the LAP pathway in mice has given additional insight into the importance of this process during infection with *A. fumigatus*. Mice lacking autophagy-related gene (Atg)7 manifest an increased fungal burden and pro-inflammatory cytokine levels during infection [42]. While β-glucan on the surface of *A. fumigatus* induces the signalling cascades for LAP, the masking of β-glucan is a viable immune-evasion strategy. Conidia are decorated with hydrophobins, thus blocking recognition by Dectin-1. Upon germination of *A. fumigatus*, β-glucan is unmasked, and LAP is initiated. Consequently, LC3-II is recruited to the fungus-containing phagosome. During infection of human monocytes with *A. fumigatus,* recruitment of LC3-II is dependent on reactive oxygen species (ROS) production by nicotinamide adenine dinucleotide phosphate (NADPH) oxidase [43]. Patients suffering from chronic granulomatous disease (CGD) have a mutation in the NADPH complex affecting their ability to produce reactive oxygen species (ROS) and display a defective immune response to infection with *A. fumigatus*, marked by poor recruitment of LC3-II to the phagosome. Regarding mice harboring the CGD mutation, the immunosuppressive drug, Anakinra, normalizes inflammasome activity and the number of LC3-II positive cells in *Aspergillus* infection [44]. Akoumianaki et al., [45] have shown that *A. fumigatus* uses cell wall melanin to functionally block LAP; similar to the way CGD affects patients. Melanin selectively interferes with ROS production by interfering with the initial assembly of the NADPH oxidase complex, thus inhibiting killing of the pathogen and advocating the development of intracellular infections. Concerning antifungal immune responses of dendritic cells (DCs) against *A. fumigatus,* hypoxia-inducible factor-1α (HIF-1α) modulates cytokine release and the metabolic profile and is partly dependent on stabilization via Dectin-1 [46].

### 3.2. Candida Albicans

Yeasts of *C. albicans* induce formation of microtubule-associated protein 1A/1B-light chain 3 (LC3) to the phagosome membrane, while there is not enough LC3 recruited to completely cover its surface [47]. This suggests that there is no immediate killing of internalized *Candida* spores via autophagy or LAP as LC3-II conjugation is critical for the successful fusion with the lysosome. Seen in primary macrophages (MΦ), as well as macrophage cell lines, only low levels of LC3 are observed in an infection with viable *C. albicans* [48]. When MΦ are exposed to beads coated with β-glucan, however, robust recruitment of LC3 transpires. Thus, it is reasonable to speculate that viable *C. albicans* yeasts do not exhibit a sufficient level of β-glucan on their surface to provoke vigorous LC3 recruitment. Yet, autophagy and LAP are critical for the immune response against infection with *C. albicans*. The importance of autophagy or LAP has been established; mice lacking autophagy-related gene (Atg)5 have a deleterious outcome in systemic infections with *C. albicans* [49]. This effect appears to be independent of changes in phagocytosis or killing of *C. albicans,* since the organism is killed by Atg knock-out cell lines comparable to controls [50]. Hypoxia-inducible factor-1α (HIF-1α) has been shown to be important for priming of the innate immune system via cathelicidin antimicrobial peptide LL-37 during infections with *Candida albicans* [51,52].

### 3.3. Histoplasma Capsulatum

Regarding *Histoplasma capsulatum,* the implication of LC3-associated phagocytosis (LAP) is still unclear as regulation of the autophagic host response is independent of Rubicon, a key regulator of LAP [53]. Upon infection, yeasts are ingested by innate immune cells. Macrophages (MΦ), the primary effector cells, permit yeast replication, while dendritic cells (DCs) kill the pathogen [54]. Killing of *H. capsulatum* yeasts by MΦ may be influenced by hypoxia-induxible factor-1α (HIF-1α). Concomitant with HIF-1α expression, yeasts provoke an increase in mitochondrial respiration and glycolysis in MΦ [37]. Ingestion of yeast cells is accompanied by microtubule-associated protein 1A/1B-light chain 3 (LC3)-II recruitment to the phagosome [55]. Enhancement of the HIF-1α signal by prolyl hydroxylase inhibitors arm MΦ to degrade intracellular yeasts; using image flow cytometry with antibodies for LC3II and labeled *H. capsulatum* yeasts, this change was linked to a reduction in LC3II in phagosomes. [54]. Survival of *H. capsulatum* in LC3-II positive phagosomes indicates that autophagy or an autophagy-like mechanism may provide a beneficial niche for the fungus. This notion was supported by experiments that applied autophagy inhibitors to infected MΦ, reporting an increase in the fungal burden compared to MΦ with intact autophagy [55]. To conclude, with the idea that autophagy provides a beneficial niche, the inhibition of autophagy lead to the degradation of *H. capsulatum*.

### 3.4. Cryptococcus Neoformans

Similar to β-glucan masking *in Aspergillus fumigatus*, *Cryptococcus neoformans* yeasts use polysaccharides, glucuronoxylomannan, and galactoxylomannan to mask β-glucan and recognition by the host immune system via Dectin-1 [56]. This immune evasion seems to be selective, as studies have shown that autophagy supports intracellular survival of *C. neoformans.* Pharmacological inhibition of autophagy in mice reduced the fungal burden with *C. neoformans*, while survival of the host was not altered [48]. Recruitment of autophagy-related gene (Atg)5 and Atg9 to the phagosome has been reported, with Ribonucleid acid interference (RNAi)-knockdowns of these proteins reducing fungal growth inside infected murine macrophages (MΦ) [57]. These findings have been further supported by reports that show increased fungal growth inside MΦ following knockdowns of the autophagy initiation complex [58]. It is possible that a receptor other than Dectin-1 is responsible for recognition of *C. neoformans* and initiation of LC3-asspciated phagocytosis (LAP). It has been shown that fragment crystallizable (Fc)-receptor activation triggers recruitment of microtubule-associated protein 1A/1B-light chain 3 (LC3) to the phagosome [59]. This is supported by a study from Nikola et al., [48] that shows differences in the recruitment pattern of LC3 the between antibody-mediated and opsonin-mediated autophagy of *C. neoformans*. Besides the possibility that *C. neoformans* is dependent on a functioning autophagic machinery, there is evidence that it is dependent on various Atgs beyond their core function in autophagy [60].

## 4. Conclusions

**Figure 1:**
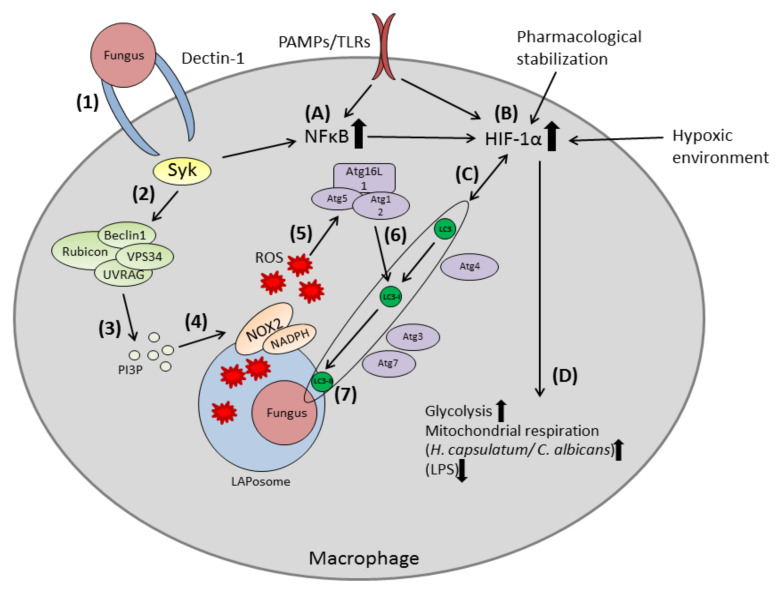
Overview of the LAP pathway in macrophages during fungal infection. (**1**) Pattern recognition receptors (PPRs) on macrophages (Dectin1) recognize fungal pathogen and initiates phagocytosis. (**2**) The Dectin1/Syk kinase complex recruits the PI3K-complex to the LAPosome. (**3**) The PI3K-complex generates PI3P, which localizes to the LAPosome. (**4**) PI3K, together with Rubicon, stabilizes the NOX2-complex, followed by assembly of the NOX2 NADPH oxidase complex. (**5**) ROS production leads to recruitment of the LC3-conjugation complex (Atg5/Atg12/Atg16L1). (**6**) Atg3, Atg4 and Atg7 form LC3-II from LC3. (**7**) Lipidation of LC3-II to the LAPosome and maturation of the LAPosome. Maturation is followed by fusion with the lysosome and killing of fungal pathogens. (**A**) The Syk-pathway simultaneously increases NFκB (**B**) HIF-1α is stabilized by increased NFκB, hypoxia or pharmacological treatment. (**C**) HIF-1α is inversely regulated to LC3-II. (**D**) HIF-1α upregulates glycolysis and mitochondrial respiration during infection with *C. albicans* or *H. capsulatum*.

Over a long period, autophagy was understood as a self-catabolic process to counteract nutritional starvation. The ability of canonical autophagy and LC3-associated phagocytosis (LAP) to recognize and clear fungal infections, however, clearly shows the potential a better understanding of autophagy has to fight against infectious pathogens. During fungal infections, canonical autophagy or LAP are activated as a first line of defense against the intruders. Even though the function of LAP in antifungal immunity is not fully understood, it is clear that it plays a vital role in the modulation of host immune responses. It is crucial to not only understand the molecular variations of autophagy and LAP during fungal infections but, also, the secondary effects of autophagy such as metabolic shifts or the presentation of fungal antigens. Like other pathogens, fungi have to overcome nutritional starvation during infection and might use the phagosome as a niche for their survival. It has become clear that fungal pathogens are often reliant on their interaction and modulation of the autophagy pathways. Here, hypoxia-inducible factor-1α (HIF-1α), linking autophagy/LAP to the modulation of metabolism, therefore, should be of special interest (Figure 1).

It has already been shown in *H. capsulatum* that modulation of HIF-1α can have a positive effect on the clearance of infection in MΦ. Though there is only little data on the role of HIF-1α as part of the antifungal host response during infection with other fungi, HIF-1α also has been shown to interact with autophagy in various other challenges, and to play a role in apoptosis. HIF-1α promotes chondrocyte survival when challenged for apoptosis [61]. Gene knockdowns of HIF-1α increased survival of adherent-invasive *Escherichia coli* in intestinal epithelial cells [62] and Lu et al., [63] have shown in SH-SY5Y cells that HIF-1α-mediated autophagy was needed for protection against hypoxic brain injuries. Data from both HIF-1α in antifungal immunity and its role in other diseases suggests HIF-1α is a promising target for further research. Once we understand the interactions and modulations of HIF-1α and autophagy, successful therapies targeting the autophagy pathways can be found to help boost our innate immune response or deprive fungal pathogens of their survival strategies.
